# Prognostic and Biological Significance of MicroRNA-127 Expression in Human Breast Cancer

**DOI:** 10.1155/2014/401986

**Published:** 2014-11-12

**Authors:** Shaohua Wang, Hanjun Li, Jingjie Wang, Dan Wang, Anlong Yao, Qiurong Li

**Affiliations:** Department of General Surgery, Jinling Hospital, School of Medicine, Nanjing University, 305 Zhongshan East Road, Nanjing, Jiangsu 210002, China

## Abstract

The purpose of this study was to determine the expression of miR-127 and analyze its prognostic and biological significance in breast cancer (BC). A quantitative reverse transcription PCR assay was performed to detect the expression of miR-127 in 15 pairs of BC and corresponding noncancerous tissues. The expression of miR-127 was detected in another 110 BC tissues and its correlations with clinicopathological factors of patients were examined. Univariate and multivariate analyses were performed to analyze the prognostic significance of miR-127 expression. The effects of miR-127 expression on malignant phenotypes of BC cells and its possible molecular mechanisms were further determined. miR-127 was significantly downregulated in BC tissues, and low miR-127 expression was significantly correlated with lymph node metastasis and advanced clinical stage. Patients with low miR-127 showed poorer overall survival than those with high miR-127. Multivariate analyses indicated that status of miR-127 was an independent prognostic factor for patients. Functional analyses showed that upregulation of miR-127 significantly inhibited growth, enhanced apoptosis, and reduced migration and invasion in BC cells by targeting the protooncogene BCL-6. Therefore, miR-127 may be a potential biomarker for predicting the survival of BC patients and might be a molecular target for treatment of human BCs.

## 1. Background

Breast cancer (BC) accounts for 22.9% of all cancers in women around the world. Survival rates in the Western world are increasing, but those in developing countries are still poor [1]. Breast carcinogenesis is a multistep process characterized by genetic and epigenetic alterations that influence key cellular pathways involved in growth and development [2]. Thus, elucidation of the molecular mechanisms involved in BC development will be helpful to exploit potential, molecular, diagnostic, and prognostic markers.

MicroRNAs (miRNAs), a class of small noncoding RNAs, function posttranscriptionally through imperfect base pairing with specific sequences in the 3′ untranslated regions (UTRs) of target mRNAs leading to transcript degradation or translational inhibition [3, 4]. Increasing evidence shows that deregulation or altered expression of miRNAs is associated with tumorigenesis and metastasis [5, 6]. Recently, the association of miRNAs with human BCs was reported [7]. Chang and his colleagues confirmed the aberrant expression of 22 miRNAs playing important roles in breast carcinogenesis by regulating the mitogen-activated protein kinase (MAPK) signaling pathway. Thus, these miRNA candidates might have high potential to play critical roles in the progression of breast cancer and could potentially serve as targets for future therapy [8]. By microarray and Northern blot analyses, Iorio and his colleagues showed that miRNAs are aberrantly expressed in human breast cancer and the overall miRNA expression could clearly separate normal versus cancer tissues, with the most significantly deregulated miRNAs being mir-125b, mir-145, mir-21, and mir-155 [9]. Moreover, some miRNAs were reported to be correlated with proliferation, apoptosis, and chemo- or radioresistance of BC cells [10–13]. Here, the focus is on miR-127. miR-127 has been reported to function as a tumor suppressor in a variety of human cancers, including BC. By analysis of the global expression profile of miRNAs in primary breast cancer and normal adjacent tumor tissues (NATs), Yan et al. showed that seven miRNAs (miR-497, miR-31, miR-355, miR-320, mir-140, miR-127, and miR-30a-3p) were downregulated more than twofold in BC tissue compared with normal adjacent tissues [14]. Also, Chen and his colleagues reported that miR-127 could regulate proliferation and senescence of breast cancer cells [15]. However, the clinicopathological and prognostic values of miR-127 in BC and its effects on migration and invasion of BC cells need to be further elucidated.

In this study, we performed qRT-PCR assay to detect expression of miR-127 and analyze its associations with clinicopathological factors and prognosis of BC patients. In addition, the effects of miR-127 on phenotypes of BC cells were determined. Here, we show that low-miR-127 expression may be an independent poor prognostic factor for BC patients. Also, restoration of miR-127 significantly inhibits growth, induces apoptosis, and reduces migration and invasion of BC cells by targeting pro-oncogene (BCL-6).

## 2. Materials and Methods

A total of 110 BC tissues, 15 pairs of BC, and corresponding noncancerous breast tissues were collected directly from surgery after removal of the necessary amount of tissue for routine pathology examination at the Department of Pathology, Jinling or Xijing Hospital, between 2003 and 2005. The tissues were immediately frozen in liquid nitrogen and stored at −80°C until use. None of the patients recruited in this study had undergone preoperative chemotherapy or radiotherapy. The characteristics of patients were shown in Table 1. Informed written consent was obtained from all patients. The tumors were frozen at −80°C in a guanidinium thiocyanate solution. The Chinese Medical Association Society of Medicine's Ethics Committee approved all aspects of this study in accordance with the Helsinki Declaration.

The human breast cancer cell lines MCF-7 and SK-BR-3 were purchased from the American Type Culture Collection (Rockville, MD). All cell lines were grown in RPMI 1640 medium with 10% fetal bovine serum at 37°C in a humidified atmosphere of 95% air and 5% CO_2_.

Total RNA isolation from tissues was performed using mirVana miRNA Isolation Kit (Applied Biosystems/Ambion, Austin, TX, USA) according to the manufacturer's instruction. The cDNA was synthesized from 5 ng of total RNA by using the TaqMan miRNA reverse transcription kit (Applied Biosystems, Foster City, CA), and the expression levels of miR-127 were quantified by using miRNA-specific TaqMan MiRNA Assay Kit (Applied Biosystems). qRT-PCR was performed by using the Applied Biosystems 7500 Sequence Detection System. The expression of miRNA was defined based on the threshold cycle (Ct), and relative expression levels of miR-127 were calculated as 2^−[(Ct of miR-127)−(Ct of U6)]^ after normalization with reference to expression of U6 small nuclear RNA.

For ectopic expression of miR-127, the pGCMV/miR-127 or pGCMV/miR-NC vectors were purchased from GenePharm (Shanghai, China). DNA template oligonucleotides targeting BCL-6 gene (GenBank accession number NM_001706) were designed and synthesized as follows: shBCL-6 (sense, 5′-*GATCCGTATATACCCGTACAACGTTTCAAGAGATCGTTGTACGGGTATATACTTTTT- TGTCGACA*-3′) and a nonspecific siRNA, shcontrol (sense, 5′-*GATCC GGTAACAAATTCCAT-AATTCAAGAGATTATGGAATTTGTTACCTTTTTTGTCGACA-*3′). All of the above were inserted into the* Bam*HI and* Hind*III sites of pSilencer4.1-CMVneo plasmid (Ambion, USA). The resulting plasmids were named pSil/shBCL-6 and pSil/shcontrol, respectively. The transfections were performed using Lipofectamine 2000 (Invitrogen, USA) according to the instructions provided by the manufacturer. The cells were transfected with those recombinant DNA vectors containing a G418 selection marker and were selected G418 (Sigma) at 500 mg/mL for 4 weeks. Then, single clones were obtained and maintained in G418 with concentration of 200 mg/mL.

Cells or tissues were washed with cold phosphate-buffered saline solution, and total proteins were extracted in the extraction buffer (150 mM sodium chloride; 50 mM Tris hydrochloride, pH 7.5; 1% glycerol; and 1% non-idetp-40 substitute solution). Equal amounts of protein (15 *μ*g per lane) from the treated cells were loaded and separated on an 8% sodium dodecyl sulfate (SDS) polyacrylamide gel and then electroblotted onto a nitrocellulose membrane, blocked by 5% skim milk, and probed with the antibodies to BCL-6, cleaved caspase-3, total caspase-3, cleaved PARP, total PARP, and GAPDH (Santa Cruz Biotechnology, Santa Cruz, CA, USA), followed by treatment with secondary antibody conjugated to horseradish peroxidase. The proteins were detected by the enhanced chemiluminescence system and exposed to X-ray film.

The cell viability was measured by MTT assay (Sigma, USA). In brief, the cells were seeded separately in 96-well plates at a density of 6.0 × 10^3^cells/200 *μ*L/well. 48 h later, 20 *μ*L MTT 5 mg/mL was added to each well, and the plates were then incubated for 4 h at 37°C. The culture media were removed; 200 *μ*L DMSO was added and incubated for 0.5 h at 37°C. The protracted cell growth curves were applied to absorbance (A) at 490 nm using VersaMax microplate reader (Molecular Devices, Sunnyvale, CA).

A total of 0.5 × 10^3^ stably transfected cells were plated in 10 cm culture dishes. After 14 days, cells were fixed with methanol and stained with 0.1% crystal violet. Visible colonies were manually counted.

The cells were harvested and washed in cold PBS. Then, Annexin V and PI staining were carried out using the Annexin V-FITC Apoptosis Detection Kit (BD Biosciences) following the manufacturer's protocol. After a 20 min incubation in the dark at room temperature, the cells were immediately analyzed by a FACScan flow cytometer (Becton Dickinson, Franklin Lakes, NJ). Each assay was performed in triplicate.

The cells were seeded into 24-well tissue culture plates. 48 h later, an artificial homogenous wound was created onto the monolayer with a sterile plastic 100 *μ*L micropipette tip. After wounding, the debris was removed by washing the cells with serum-free medium. Migration of cells into the wound was observed at different time points. Cells that migrated into the wounded area or cells with extended protrusion from the border of the wound were visualized and photographed under an inverted microscope.

For invasion assay, 1.0 × 10^3^ cells were plated in the top chamber onto the Matrigel coated membrane (24-well insert; pore size, 8 *μ*m; Corning Costar). Each well was coated freshly with Matrigel (60 mg; BD Bioscience) before the invasion assay. Cells were plated in medium without serum or growth factors, and medium supplemented with serum was used as a chemoattractant in the lower chamber. The cells were incubated for 48 hours and cells that did not invade through the pores were removed by a cotton swab. Cells on the lower surface of the membrane were fixed with methanol and stained with crystal violet. The number of cells invading through the membrane was counted under a light microscope.

To testify the validation of BCL-6 as a direct target of miR-127, we performed miRNA target luciferase reporter assay using a pLUC target reporter plasmid containing BCL-6/3′-UTR (Genecoepia, Rockville, MD) (pLUC/BCL-6/3′-UTR-wt). Additionally, we generated mutant BCL-6/3′-UTR reporter construct by site-directed mutagenesis in the putative target site of miR-127 in the wild-type BCL-6/3′-UTR (pLUC/BCL-6/3′-UTR-mut) using Stratagene QuikChange Site-Directed Mutagenesis Kit (Stratagene, Heidelberg, Germany). The cells were transiently cotransfected for 24 h with reporter plasmids (200 ng) and 100 nM of miR-409-3p mimics or inhibitor and harvested in reporter lysis buffer. Both firefly luciferase and Renilla luciferase activities were measured using the Dual-Luciferase assay kit (Promega, Madison, WI) according to the manufacturer's instructions. The luciferase activity normalized against protein concentration was expressed as a ratio of firefly luciferase to Renilla luciferase unit.

All statistical analyses were carried out using the SPSS 17.0 software package (SPSS, Chicago, IL, USA). The data were presented as the mean ± SD. The chi-squared test was used to investigate the significance of miR-127 expression as being correlated with clinicopathologic factors in BC. The disease-free survival (DFS) and overall survival (OS) curves were plotted using the Kaplan-Meier method and were evaluated for the statistical significance using a log-rank test. The significance of different variables with respect to survival was analyzed using the univariate and multivariate Cox proportional hazards model. Differences were considered statistically significant when *P* < 0.05.

## 3. Results

### 3.1. miR-127 Was Significantly Downregulated in BC Tissues

**Figure 1:**
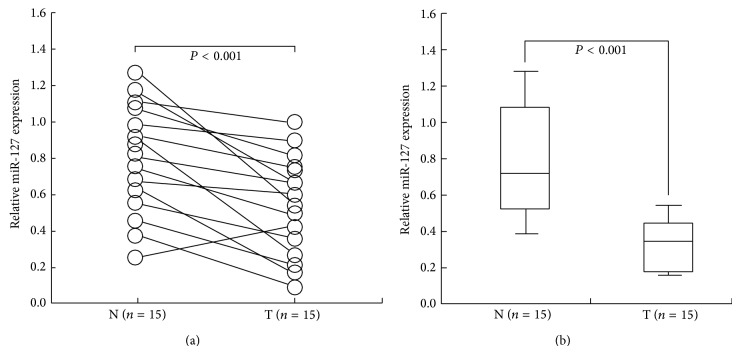
qRT-PCR detection of miR-127 expression in BC tissues. (a) qRT-PCR detection of mature miR-127 expression in 15 pairs of BC (T) and corresponding noncancerous breast tissues (N) (*P* < 0.001). (b) Calculating the mean level of miR-127 in 15 pairs of BC and corresponding noncancerous tissues, respectively (*P* < 0.001). The mean and standard deviation of expression levels relative to U6 expression levels are shown and are normalized to the expression in the normal tissue of each matched pair. Each experiment was performed at least in triplicate.

To determine the status of miR-127 expression in BC, a qRT-PCR assay was performed to determine the expression of miR-127 in 15 pairs of BC and corresponding noncancerous tissue samples. Results showed that the relative level of miR-127 expression in BC tissues was significantly downregulated, compared with that in corresponding noncancerous tissues (*P* < 0.001; Figures 1(a)-1(b)).

### 3.2. Correlation of miR-127 Expression with Clinicopathologic Factors of BC Patients

To further analyze the clinicopathological significance of miR-127 in BC, the expression of miR-127 was determined in another 110 cases of BC tissues. The median value of miR-127 in all BC tissues was 0.86 and was used as a cutoff value, and all patients were divided into two groups high-miR-127 expression group (≥0.86; *n* = 44) and low-miR-127 expression group (<0.86; *n* = 66). Table 1 showed the correlation between miR-127 expression and clinicopathological factors of BC patients. By statistical analyses, low-miR-127 expression was found to be significantly correlated with higher incidence of lymph node metastasis and advanced clinical stage (*P* = 0.029 and 0.004, resp.). However, there were no significant correlations between miR-127 expression and other clinicopathological factors of patients, such as age, tumor size, histological type, differentiation grade, ER, PR, and HER-2 status (*P* = 0.749, 0.500, 0.795, 0.352, 0.936, 0.340, and 0.640, resp.). These data indicated that downregulation of miR-127 might play a critical role in BC progression.

### 3.3. Correlation of miR-127 Expression with Prognosis of BC Patients

**Figure 2:**
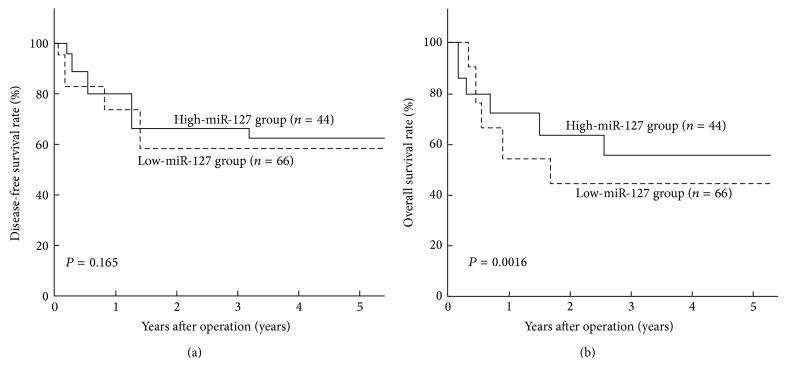
Kaplan-Meier DFS and OS curves of BC patients. (a) The 5-year DFS rate showed no difference between BC patients with low-miR-127 expression and those patients with high-miR-127 expression (*P* = 0.165). (b) The 5-year OS rate of BC patients with high-miR-127 expression was significantly higher than that of those patients with low-miR-127 expression (*P* = 0.0016). Corresponding *P* values analyzed by log-rank tests are indicated.

Kaplan-Meier analyses were performed to analyze the correlation between miR-127 expression and disease-free survival (DFS) and overall survival (OS) of BC patients. It was observed that the 5-year OS of high-miR-127 group (57.4%) was significantly higher than that of low-miR-127 group (44.8%; *P* = 0.0016) (Figure 2(b)). However, there was no significant difference between the 5-year DFS of high-miR-127 group and that of low-miR-127 group (61.4%* versus* 59.3%; *P* = 0.165) (Figure 2(a)).

### 3.4. Univariate and Multivariate Analysis of Prognostic Factors in BC Patients

Univariate and multivariate analyses of factors related to prognosis of BC patients were shown in Table 2. Multivariate regression analysis indicated that status of miR-127 expression (relative risk (RR), 1.798; 95% confidence interval (CI), 1.333–2.552; *P* = 0.038) was significantly correlated with OS of BC patients, along with HER-2 status (*P* = 0.028), lymph node metastasis (*P* = 0.007), and clinical stage (*P* = 0.025). Furthermore, multivariate regression analysis indicated that status of miR-127 expression (RR, 2.023; 95% CI, 1.437–2.855; *P* = 0.009) was an independent predictor for the prediction of OS, as well as lymph node metastasis (RR, 2.097; 95% CI, 1.005–2.506; *P* = 0.012) and clinical stage (RR, 1.676; 95% CI, 1.278–3.121; *P* = 0.006).

### 3.5. Upregulation of miR-127 Inhibits Growth, Reduces Colony Formation, and Enhances Apoptosis of BC Cells

**Figure 3:**
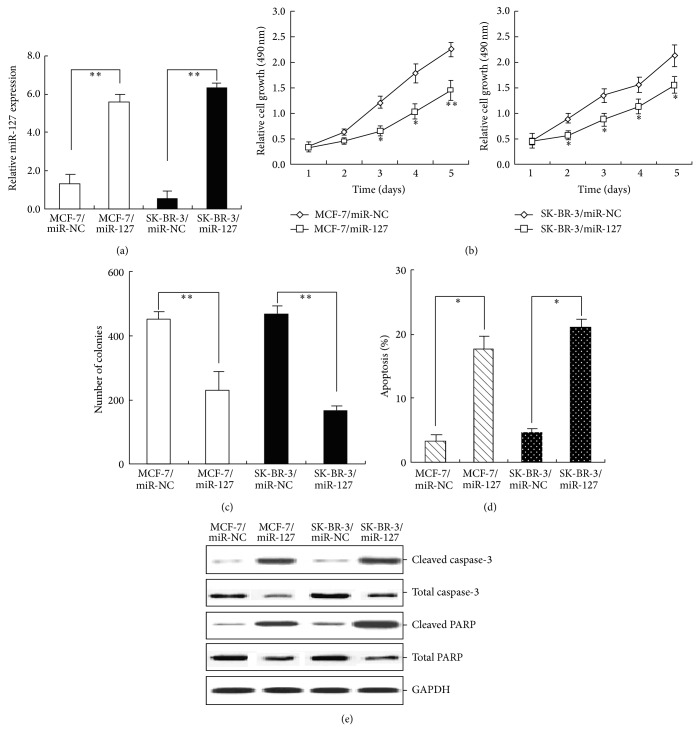
Upregulation of miR-127 expression inhibits growth, reduces colony formation, and enhances apoptosis in BC cells. (a) qRT-PCR analysis of miR-127 expression in stably transfected MCF-7/miR-127 (or MCF-7/miR-NC) and SK-BR-3/miR-127 (SK-BR-3/miR-NC) cells. U6 was used as an internal control. (b) MTT analysis of growth in MCF-7/miR-127 (or MCF-7/miR-NC) and SK-BR-3/miR-127 (or SK-BR-3/miR-NC) cells. (c) The colony formation assay was performed as described in Materials and Methods section. The number of colonies was counted and compared. (d) Flow cytomeric detection of apoptosis in MCF-7/miR-127 (or MCF-7/miR-NC) and SK-BR-3/miR-127 (or SK-BR-3/miR-NC) cells. (e) Western blot analysis of the expression levels of cleaved caspase-3, total caspase-3, cleaved PARP, and total PARP proteins. GAPDH was used as an internal control. Each experiment was performed at least in triplicate. ^*^ *P* < 0.05, ^**^ *P* < 0.01 versus control.

To analyze the effects of miR-127 expression on malignant phenotypes of BC cells, pGCMV/miR-127 vector or control vector (pGCMV/miR-NC) was stably transfected into BC cells (MCF-7 and SK-BR-3), which was named MCF-7/miR-127 (or MCF-7/miR-NC) and SK-BR-3/miR-127 (or SK-BR-3/miR-NC), respectively. qRT-PCR was used to detect the expression of miR-127 in MCF-7/miR-127 and MCF-7/miR-NC, respectively. Compared with that in control cells, the relative level of miR-127 expression in MCF-7/miR-127 and SK-BR-3/miR-127 cells was significantly increased by about 423.8% and 508.4%, respectively (*P* < 0.01; Figure 3(a)). Results from MTT assays indicated that upregulation of miR-127 could significantly inhibit growth of BC cells (Figure 3(b)). The capacity of colony formation in MCF-7/miR-127 and SK-BR-3/miR-127 cells was significantly reduced, in comparison with that in the control cells (*P* < 0.05; Figure 3(c)). Furthermore, the apoptotic rate of MCF-7/miR-127 or SK-BR-3/miR-127 cells was significantly increased by about 14.5% and 16.6%, respectively (Figure 3(d)). The expression of cleaved caspase-3 and PARP proteins was significantly increased in MCF-7/miR-127 or SK-BR-3/miR-127 cells in comparison with control cells (Figure 3(e)). These data showed that upregulation of miR-127 could inhibit growth and reduced the capacity of colony formation in BC cells by enhancing caspase-3-dependent apoptosis.

### 3.6. Upregulation of miR-127 Significantly Inhibits Migration and Invasion of BC Cells

**Figure 4:**
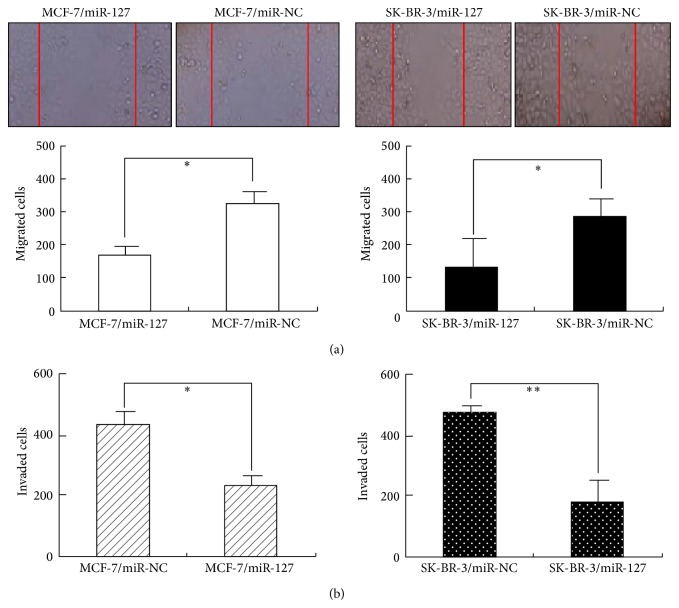
Upregulation of miR-127 inhibits migration and invasion in BC cells. (a) Scratch wound healing analysis of migration in BC cells. MCF-7/miR-127 (or MCF-7/miR-NC) and SK-BR-3/miR-127 (or SK-BR-3/miR-NC) cells were grown for 3 days and subjected to the wound healing assay. Magnification ×100. (b) Transwell invasion analysis of invasion in BC cells. MCF-7/miR-127 (or MCF-7/miR-NC) and SK-BR-3/miR-127 (or SK-BR-3/miR-NC) cells were grown for 3 days and subjected to the Transwell invasion assay. Magnification ×200. Each experiment was performed at least in triplicate. ^*^ *P* < 0.05, ^**^ *P* < 0.01 versus control.

Next, we explored the effects of miR-127 expression on migration and invasion of BC cells. Scratch wound healing assay indicated that the migration of MCF-7/miR-127 or SK-BR-3/miR-127 cells was markedly decreased in comparison with that of MCF-7/miR-NC or SK-BR-3/miR-NC cells (*P* < 0.05; Figure 4(a)). Similarly, Matrigel invasion assays indicated that the invasion of MCF-7/miR-127 or SK-BR-3/miR-127 cells was significantly reduced in comparison with that of MCF-7/miR-NC or SK-BR-3/miR-NC cells (*P* < 0.05; Figure 4(b)). Thus, upregulation of miR-127 could reduce the capacity of migration and invasion in BC cells.

### 3.7. BCL-6 Was a Direct Target of miR-127

**Figure 5:**
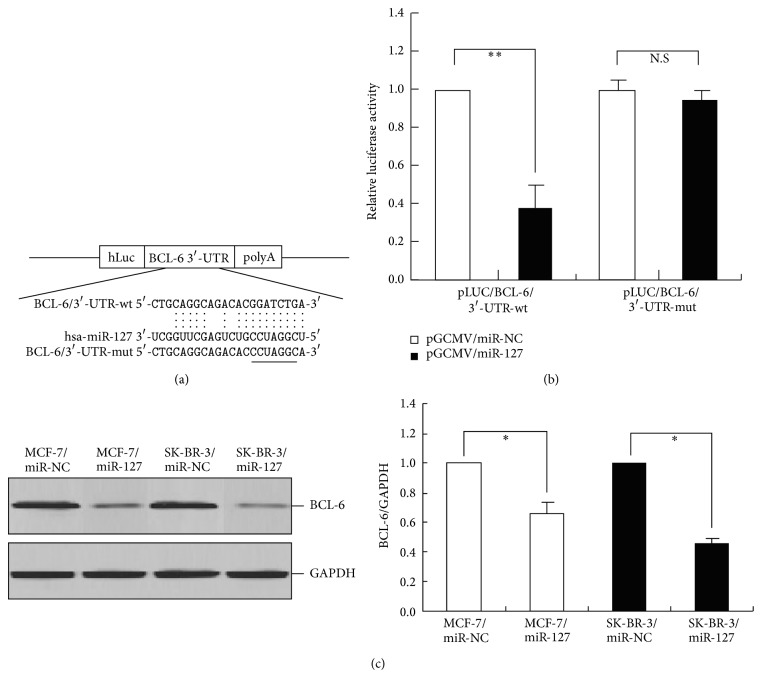
miR-127 binds to the 3′-UTR of BCL-6 mRNA. (a) A human BCL-6/3′-UTR fragment containing wild-type or mutant miR-127-binding sequence was cloned downstream the luciferase reporter gene in pLUC-luc vector. (b) pLUC-luc vector contains BCL-6/3′-UTR-wt or BCL-6/3′-UTR-mut and pGCMV/miR-127 or control pGCMV/miR-NC were cotransfected into HEK293T cells. Cells lysates were prepared after 48 h for measuring luciferase activity, which was normalized to Renilla luciferase activity. (c) Western blot analysis of BCL-6 protein expression in MCF-7/miR-127 (or MCF-7/miR-NC) and SK-BR-3/miR-127 (or SK-BR-3/miR-NC) cells. GAPDH was used as an internal control. Data were presented as mean ± SEM (*n* = 3). ∗ or ∗∗ indicates *P* < 0.05 or <0.01, respectively.

To explore how miR-127 contributes to the malignant phenotypes of BC cells, we searched for the potential regulatory targets of miR-127 using prediction tools, including miRanda, PicTar, and TargetScan. Although hundreds of different targets were predicted, those genes involved in the pathogenesis of human cancers may be the relevant targets with respect to the biological functions of miR-127. Of these genes, BCL-6 is regarded as a proto-oncogene that was originally characterized as a regulator of B-lymphocyte development and growth and has been implicated in many human malignancies, including breast cancer. To obtain further direct evidence that BCL-6 is a target of miR-127, we investigated the binding site of miR-127 in the 3′-UTR sequence of BCL-6 mRNA. We constructed a luciferase reporter (pLUC/BCL-6/3′-UTR-wt) in which the nucleotides of the BCL-6-3′-UTR complementary to miR-127 (nt 3057–3080) were inserted into the pLUC vector, and we also generated a mutant reporter (pLUC/BCL-6/3′-UTR-mut), in which the first six nucleotides in the miR-127 seed region complementary sites were mutated (Figure 5(a)). 48 h after pGCMV/miR-127 or pGCMV/miR-NC were cotransfected with pLUC/BCL-6/3′-UTR-wt or pLUC/BCL-6/3′-UTR-mut into human HEK293T cells, the luciferase activity was determined. Results showed that the luciferase activity in the pLUC/BCL-6/3′-UTR-wt-transfected cells was significantly reduced compared to the luciferase activity in the pLUC/BCL-6/3′-UTR-mut-transfected cells (*P* < 0.01) (Figure 5(b)). Next, we analyzed the effect of miR-127 on the expression of BCL-6 by Western blotting to detect the expression of BCL-6 protein in MCF-7/miR-127 or SK-BR-3/miR-127 and their respective control cells. It was observed that upregulation of miR-127 could significantly inhibit the expression of BCL-6 protein in MCF-7 and SK-BR-3 cells (Figure 5(c)). These data suggest that BCL-6 might be a direct target of miR-127.

### 3.8. Silencing of BCL-6 Reverses Malignant Phenotypes of BC Cells

**Figure 6:**
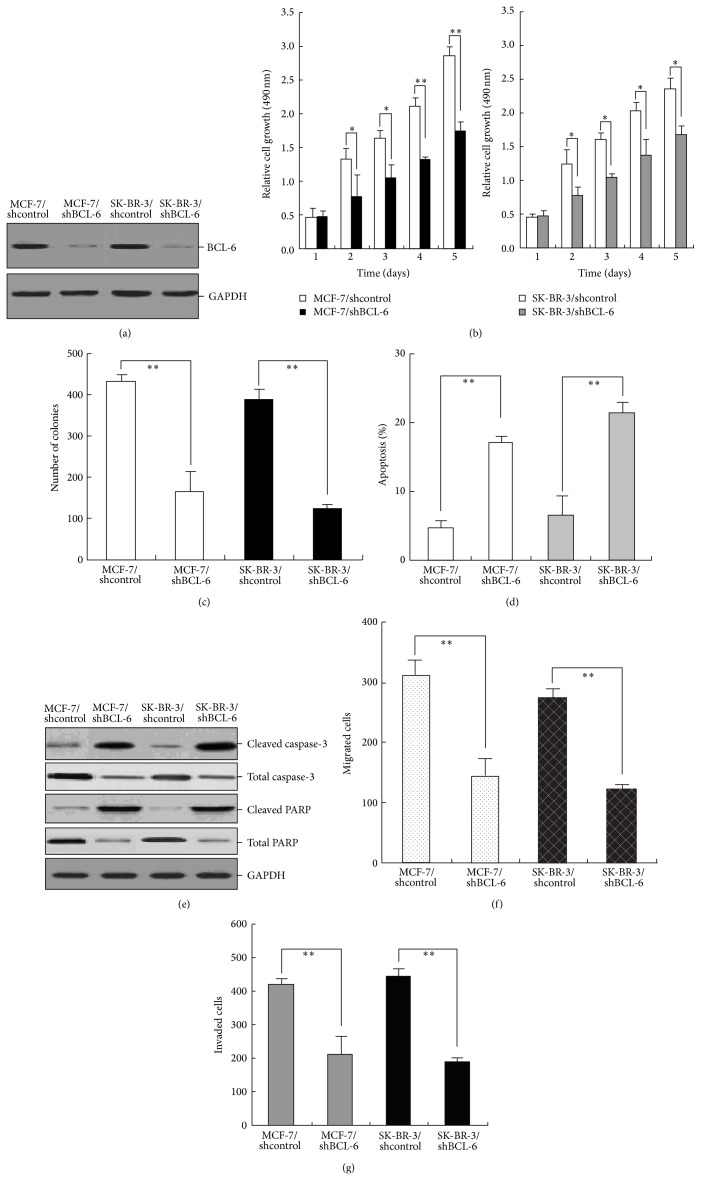
Silencing of miR-127 reverses malignant phenotypes of BC cells. (a) Western blot analysis of BCL-6 protein expression in stably transfected MCF-7/shBCL-6 (or MCF-7/shcontrol) and SK-BR-3/shBCL-6 (or SK-BR-3/shcontrol) cells. GAPDH was used as an internal control. (b) MTT analysis of growth in MCF-7/shBCL-6 (or MCF-7/shcontrol) and SK-BR-3/shBCL-6 (or SK-BR-3/shcontrol) cells. (c) The colony formation assay was performed as described in Materials and Methods section. The number of colonies was counted and compared. (d) Flow cytomeric detection of apoptosis in MCF-7/shBCL-6 (or MCF-7/shcontrol) and SK-BR-3/shBCL-6 (or SK-BR-3/shcontrol) cells. (e) Western blot analysis of the expression levels of cleaved caspase-3, total caspase-3, cleaved PARP, and total PARP proteins in MCF-7/shBCL-6 (or MCF-7/shcontrol) and SK-BR-3/shBCL-6 (or SK-BR-3/shcontrol) cells. GAPDH was used as an internal control. (f) Scratch wound healing analysis of migration in MCF-7/shBCL-6 (or MCF-7/shcontrol) and SK-BR-3/shBCL-6 (or SK-BR-3/shcontrol) cells. (g) Transwell invasion analysis of invasion in MCF-7/shBCL-6 (or MCF-7/shcontrol) and SK-BR-3/shBCL-6 (or SK-BR-3/shcontrol) cells. Each experiment was performed at least in triplicate. ^*^ *P* < 0.05, ^**^ *P* < 0.01 versus control.

**Figure 7:**
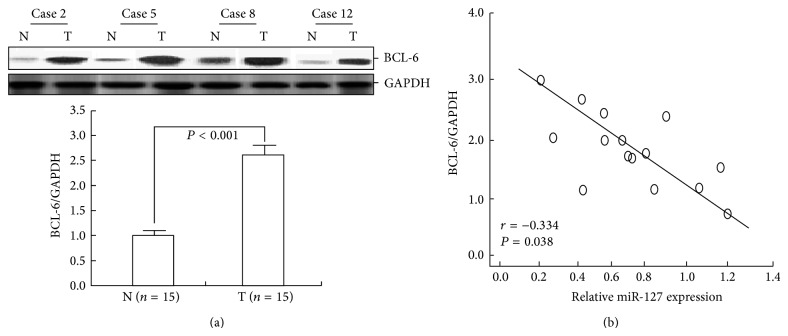
The expression of BCL-6 protein was significantly upregulated and inversely correlated with miR-127 expression in BC tissues. (a) Western blotting detection of BCL-6 protein expression in 15 pairs of BC (T) and corresponding noncancerous tissues (N). GAPDH was used as an internal control. (b) A statistically significant inverse correlation between miR-127 and BCL-6 protein expression levels in BC tissues (*n* = 15; Spearman's correlation analysis, *r* = −0.334; *P* = 0.038). Results represent the average of three independent experiments (mean ± SD). Corresponding *P* values analyzed by Spearman correlation test are indicated.

To further investigate the roles of BCL-6 in BC progression, shRNA vector targeting BCL-6 (pSil/shBCL-6) was stably transfected into MCF-7 cells and the effects of BCL-6 expression on malignant phenotypes of BC cells were determined. Compared with that in MCF-7/shcontrol or SK-BR-3/shcontrol cells, the expression of BCL-6 protein in MCF-7/shBCL-6 or SK-BR-3/shBCL-6 cells was significantly reduced (Figure 7(a)). MTT and colony formation assays indicated that downregulation of BCL-6 could lead to the decreased activities of growth and colony formation in BC cells (Figures 6(b)-6(c)). At the same time, it was also observed that silencing of BCL-6 could enhance apoptosis of MCF-7 cells by activation of caspase-3 (Figure 6(d)). Scratch wound healing and Transwell invasion assays showed that the migration and invasion of MCF-7/shBCL-6 and SK-BR-3/shBCL-6 cells were significantly reduced in contrast to control cells (Figures 6(e)-6(f)). Therefore, silencing of BCL-6 could mimic the effects of miR-127 upregulation on malignant phenotypes of BC cells.

### 3.9. Expression of miR-127 in BC Tissues Was Inversely Associated with BCL-6 Expression

A Western blotting assay was performed to detect the expression of BCL-6 protein in the above-mentioned 15 pairs of BC and corresponding noncancerous tissues. Results showed that the mean expression level of BCL-6 protein in BC tissues was significantly higher than in noncancerous tissues (*P* < 0.001; Figure 7(a)). Next, we investigated whether BCL-6 protein expression was inversely correlated with levels of miR-127 in BC tissues. A total of 15 BC tissues were analyzed for the expression levels of BCL-6 and for miR-127 expression. A statistically significant inverse correlation was observed between BCL-6 and miR-127 (*r* = −0.334; *P* = 0.038, Pearson's correlation; Figure 7(b)). These data suggested that downregulation of miR-127 was inversely correlated with upregulation of BCL-6 in BC tissues.

## 4. Discussion

In the present study, we showed that miR-127 was significantly downregulated in BC tissues in comparison with corresponding noncancerous tissues. Also, low-miR-127 expression was observed to be significantly correlated with higher incidence of lymph node metastasis and advanced clinical stage of BC patients. Additionally, the OS of patients with low miR-127 was significantly lower than that of those patients with high miR-127, and multivariate analysis indicated that status of miR-127 was an independent factor for predicting the survival of BC patients. In addition, upregulation of miR-127 could inhibit growth, reduce colony formation, and enhance apoptosis of BC cells by targeting BCL-6.

The microRNA-127 (miR-127) is located within the imprinted Dlk1/Gtl2 region expressed from the maternal chromosome. miR-127 and miR-433 are transcribed from independent promoters in overlapping genomic regions, and expression of these two miRNAs is induced by estrogen related receptor gamma (ERRc) and inhibited by small heterodimer partner (SHP), a unique orphan nuclear receptor and transcriptional repressor [16, 17]. The first study indicating that miR-127 might be a putative tumor suppressor was based on findings that miR-127 levels could be induced by the chromatin-modifying drug 5′-aza-2′-deoxycytidine in a panel of human tumor cell lines including T24, HCT116, Hela, NCCIT, and Ramos cells [18]. Guo and his colleagues showed that the ectopic expression of miR-433 and miR-127 in gastric cancer cell lines inhibits cell proliferation, cell cycle progression, cell migration, and invasion by directly interacting with the mRNA encoding oncogenic factors KRas and MAPK4, respectively [19]. By next generation sequencing analysis of miRNAs, Jiang et al. reported that miR-127-3p inhibits glioblastoma proliferation and activates TGF-*β* signaling by targeting SKI (v-ski sarcoma viral oncogene homolog (avian)) [20]. Yang and his colleagues firstly elucidated a feedback regulation between miR-127 and the TGF*β*/c-Jun cascade in HCC migration via MMP13 that involves a cross talk between the oncogene c-Jun and tumor suppressor p53 [21]. Tudor-SN, as a component in RNA-induced splicing complex, was recently reported to regulate gene expression in a microRNA- (miRNA-) dependent manner. Zhao and his colleagues also found that Tudor-SN promoted metastasis and proliferation of breast cancer cells via downregulating the miR-127 expression [22], although miR-127 could regulate breast cell proliferation and senescence [15]. The clinicopathological and prognostic significance of miR-127 expression in BC is still unclear and needs to be further elucidated.

Here, we first detected the expression of miR-127 in 15 pairs of BC tissues and corresponding noncancerous tissues and showed that the relative expression level of miR-127 in BC tissues was significantly lower than that in corresponding noncancerous tissues. By statistical analysis, the expression of miR-127 was observed to be significantly correlated with advanced clinical stage and higher incidence of lymph node metastasis. Results from survival analyses suggested that the expression of miR-127 was correlated to OS but not to DFS of BC patients. Univariate and multivariate analyses indicated that status of miR-127 expression, along with lymph node metastasis and clinical stage, might be an independent prognostic factor for BC patients. To further determine the roles of miR-127 in BC development, we analyzed the effects of miR-127 on malignant phenotypes of BC cells. MTT and colony formation assays indicated that upregulation of miR-127 could significantly inhibit growth and reduce colony formation capacity of BC cells. Further researches suggested that upregulation of miR-127 could enhance apoptosis of BC cells, which might be associated with activation of caspase-3. These data indicated that miR-127 functions as a tumor suppressor in BC. As miRNAs function by targeting mRNA, understanding the mechanism of action and identifying functionally important mRNA targets of a specific miRNA will contribute to unravelling its biological function [23]. By integrating bioinformatics analysis, BCL-6 was identified as a direct functional downstream target of miR-127. Previously, Saito and his colleagues also reported specific activation of microRNA-127 with downregulation of the proto-oncogene BCL-6 by chromatin-modifying drugs in human cancer cells [18]. The human proto-oncogene BCL-6 on chromosome 3 at q27 encodes a BTB/POZ-zinc finger transcriptional repressor that is necessary for germinal center formation and is implicated in the pathogenesis of B cell lymphoma [24]. It has been reported that BCL-6 is expressed in breast cancer and prevents mammary epithelial differentiation [25]. In invasive breast cancer, protein expression of BCL-6 was observed to be associated with cyclin D1 and hypoxia-inducible factor-1-alpha (HIF-1-alpha), first suggesting that BCL-6 oncogene activation plays a role in cancers other than lymphomas [26]. Whether miRNAs play critical roles in the activation of BCL-6 needs to be investigated. A recent study has shown that BCL-6 in BC cells could be targeted by miR-339-5p [27]. One mRNA can also be regulated by many microRNAs, so BCL-6 in BC cells might be targeted by other miRNAs. By analysis of luciferase activity, miR-127 can bind with 3′-UTR sequence of BCL-6 mRNA. In concordance with the luciferase reporter results, endogenous BCL-6 protein levels were found to be downregulated by miR-127 in BC cells. At the same time, silencing of BCL-6 could mimic the effects of miR-127 upregulation on malignant phenotypes of BC cells. More importantly, the relative expression level of BCL-6 protein in BC tissues was observed to be inversely correlated with the expression level of miR-127. These data clearly suggested that BCL-6 might be a direct and functional target of miR-127 in BC cells.

Taken together, downregulation of miR-127 was significantly correlated with higher lymph node metastasis, advanced clinical stage, and poor overall survival of BC patients. Additionally, upregulation of miR-127 could significantly suppress growth, reduce colony formation, enhance apoptosis, and inhibit migration and invasion of BC cells, at least partially by targeting BCL-6. Thus, miR-127 could be a potential prognostic biomarker and therapeutic target for BC. Of course, this study has several limits. First, as the number of patients in this study is small, a larger case population is needed to confirm the prognostic value of miR-127 expression in BC. Second, further studies are needed to identify other undefined miR-127 targets, which may also affect cellular phenotypes at other levels.

## Figures and Tables

**Table 1:** The correlations between miR-127 expression and clinicopathological factors of BC patients.

Factors	Relative miR-127 expression		*P* value
	High (*n* = 44)	Low (*n* = 66)	
Age (years)			0.749
≤50	16	26	
>50	28	40	
Tumor size (cm)			0.500
≤2.0	32	44	
>2.0	12	22	
Differentiation grade			0.795
G1 + 2	13	18	
G3	31	48	
Histological type			0.352
Ductal	26	32	
Lobular	18	32	
ER status			0.936
Negative	17	26	
Positive	27	40	
PR status			0.340
Negative	20	24	
Positive	24	42	
HER-2 status			0.640
Negative	22	30	
Positive	22	36	
Lymph node metastasis			0.029^*^
Absent	26	25	
Present	18	41	
Clinical stage			0.004^*^
I + II	31	28	
III	13	38	

^*^Statistically significant difference (*P* < 0.05). ER: estrogen receptor; PR: progesterone receptor; HER-2 c-erbB-2.

**Table 2:** Univariate and multivariate analysis for OS of BC patients.

Variables	Univariate analysis		Multivariate analysis	
	RR (95% CI)	*P* value	RR (95% CI)	*P* value
Age (year)	0.665 (0.342–1.212)	0.104		
Tumor size (cm)	0.882 (0.602–1.018)	0.318		
Differentiation grade	0.912 (0.708–2.023)	0.175		
ER status	0.559 (0.512–1.022)	0.084		
PR status	0.912 (0.833–1.642)	0.322		
HER-2 status	2.921 (1.456–2.873)	0.028^*^	0.598 (0.423–0.977)	0.118
Lymph node metastasis	1.877 (1.228–3.702)	0.007^*^	2.097 (1.005–2.506)	0.012^*^
Clinical stage	2.538 (1.318–2.808)	0.025^*^	1.676 (1.278–3.121)	0.006^*^
miR-127 expression	1.798 (1.333–2.552)	0.038^*^	2.023 (1.437–2.855)	0.009^*^

^*^ *P *< 0.05. HR: hazard ratio; 95% CI: 95% confidence interval.
